# Challenges of recruitment processes to a randomized dietary trial in irritable bowel syndrome

**DOI:** 10.12688/f1000research.147710.2

**Published:** 2024-06-24

**Authors:** Bodil Roth, Bodil Ohlsson

**Affiliations:** 1 Department of Internal Medicine, Skåne University Hospital, Malmö, 20502, Sweden; 2 Clinical Sciences, Lund University, Malmö, 20502, Sweden

**Keywords:** dietary habits; dietary intervention; irritable bowel syndrome, recruitment process

## Abstract

**Background:**

Irritable bowel syndrome (IBS) is common with a global prevalence of 4%. Dietary regimes with a low content of fermentable oligo-, di-, and monosaccharides and polyol (FODMAP) or a starch- and sucrose-reduced diet (SSRD) have proven to be efficient. The aim of the present study was to describe the recruitment process for a randomized dietary trial with low FODMAP or SSRD for 4 weeks with a follow-up period of 5 months. The results of the dietary trial itself are not included in this paper but will be presented in another publication.

**Methods:**

The County of Skåne, with 1,41 million inhabitants, was used as a base to perform a dietary trial in which IBS patients, age 18-70 years, were randomized to either low FODMAP or SSRD for 4 weeks. The estimated number of IBS patients in the actual age span was approximately 32,000. The trial was announced through lectures, letters to all primary healthcare centers (n=203), social media (two campaigns), and invitations to IBS patients identified in medical records (n=744).

**Results:**

Three referrals arrived from the healthcare system, 17 patients contacted the investigators in person after receiving information from their healthcare center, and four patients contacted the investigators after recommendations from friends. Of these, 14 were enrolled in the study. From social media, 218 names were delivered, of which 93 fulfilled the study criteria and were willing to participate when contacted by the investigators (42.7%). Of the 3587 identified IBS patients in medical records in close proximity to the hospital, 744 were randomly contacted. Forty-eight patients (6.5 %) were willing to be included in the study. Thus, 155 patients with IBS were included in this study.

**Conclusions:**

The inclusion rate for dietary intervention was very low considering the large population informed about the study. Announcements on social media seem to be the best way to recruit patients for intervention.

**Trial registration:**

NCT05192603, 29/11/2021, ClinicalTrials.gov. The PRS URL is https://register.clinicaltrials.gov

## Background

Irritable bowel syndrome (IBS) is the most common functional gastrointestinal disorder (FGID), with a global prevalence of 4% according to Rome IV criteria. ^1^^,^^2^ The prevalence of any functional gastrointestinal symptom is 40% in the general population. ^2^ Apart from bowel symptoms, extraintestinal symptoms and comorbidities are common, with a great impact on quality of life. ^3^^,^^4^ Many patients with IBS do not seek medical care, but between 10% and 70% of patients are estimated to attend primary care; the prevalence depends on the country. ^5^ Individuals who met the Rome IV criteria for any FGID were more likely to have visited healthcare at any time point than individuals without these diseases. ^2^ Taken together, FGID/IBS is the most common gastrointestinal entity, leading to the most frequent healthcare visits. ^6^ Due to the great healthcare burden, these diseases lead to both direct and indirect costs for the health service and society. ^7^^,^^8^

The effect of drugs in FGID/IBS is moderate, with a therapeutic gain of 10%–15% over placebo. ^9^ Most FGID patients report food-related symptoms, ^10^ leading to dietary changes in approximately half of the patients due to their symptoms. ^11^ Accordingly, the most efficient treatment for IBS is dietary accommodation, and 50%–75% is improved by a low content of fermentable oligo-, di-, and monosaccharides and polyol (FODMAP). ^12^ A 70% effect on gastrointestinal symptoms has recently been described for a starch- and sucrose-reduced diet (SSRD) in IBS, with improved dietary intake and a healthier endocrine profile. ^13^^–^^15^ Therefore, we aimed to perform a dietary intervention to compare low FODMAP diet and SSRD diets. However, motivating individuals to change their dietary habits is challenging. The aim of the present brief report was to describe the recruitment and inclusion processes of dietary interventions for IBS. The results of the dietary trial itself will be presented in another publication.

## Methods

### Study design

The study was an open randomized, non-inferior trial, with two parallel groups, performed at the Department of Internal medicine, Skåne University Hospital. Malmö, Sweden. The study was registered at ClinicalTrials.gov (NCT05192603), date 29/11/2021. A dietary intervention was given for 4 weeks, followed by another 5 months when the participants were allowed to eat whatever they preferred, before a final appointment, corresponding to three physical appointments. Study questionnaires (30 min to complete), diary books registered on-line for 3 days, and fecal samples were collected at home before each appointment and saved for later analyses. The visit took 45 min when blood samples were drawn. The primary study outcome was to measure the responding rate concerning gastrointestinal symptoms, and secondary outcomes to measure mechanisms behind the response.

### Inclusion and exclusion criteria

The study inclusion criteria were diagnosis of IBS with abdominal pain at least weekly with a total irritable bowel syndrome severity scoring system (IBS-SSS) > 175, age 18–70 years, and ability to understand the Swedish language. ^16^ The exclusion criteria were diagnosis of celiac disease, inflammatory bowel disease (IBD), bile acid malabsorption, infectious gastroenteritis, severe gastrointestinal surgery in the past, or enteric dysmotility. Participants were excluded if they had alcohol or drug abuse, severe food allergy, current eating disturbances, or any other severe organic or psychiatric disease. Pregnancy or a low FODMAP diet, low carbohydrate and high fat diet (LCHF), gluten-free diet, or vegan diet were also excluded.

### Population base for the recruitment process

The recruitment process was initiated 13/12/2021 and was ongoing throughout the study period until study recruitment was completed 13/09/2023. The first participant was allocated 03/03/2022. The study information was directed to the entire county of Skåne, with 1,41 million inhabitants. Divided into 5-year age classes, this rendered 793,914 inhabitants between 20 and 69 years old by the end of 2021. ^17^ By calculating the prevalence of FGID (39%) and IBS (4%) in Sweden, ^2^ the County of Skåne should have 309,626 subjects with FGID and 31,757 subjects with IBS according to Rome IV in the age range of 20–69 years. ^2^ Owing to the inclusion criteria of 18–70 years, more IBS patients were expected to be available.

### Information

In all information distributed, it was clearly stated that all participants were randomized to a dietary treatment, either in the form of low FODMAP or SSRD. ^12^^,^^13^ Furthermore, information on the study design and practical issues is provided. Both groups received verbal and written dietary advice with recipes and menu suggestions to enable compliance to the diet, which they had to buy and cook themselves. Patients randomized to SSRD focused on reduction of sucrose-containing food and change from starch-rich fruits and vegetables to less starch-containing fruits/vegetables. Only one serving per day was allowed for whole‐grain bread and lesser amounts of rice and pasta was recommended. Increased intake of fish and dairy products was encouraged. ^13^ Processed food and fast food should be avoided. Participants randomized to the low FODMAP received information how to avoid or reduce intake of fructans, galacto-oligosaccharides, lactose, fructose more than glucose (e.g., honey), and polyols during the 4-week intervention. ^12^

*Lectures*

The principal investigator (PI) (BO) held two lectures at regular meetings for researchers from primary healthcare, representing several healthcare centers. The PI also held a digital lecture for primary healthcare in the whole region with invitations to all general practitioners (GP), as well as a physical lecture at the Department of Internal Medicine, Skåne University Hospital. Afterwards, the lecture was made available on the website of Region Skåne. The head of the dieticians in the region was informed about the study and forwarded the information to all dieticians. All dietitians in the environment of the PI were informed in person. The head of the Department of Gastroenterology was informed about the study, and a recommendation was stated orally and written at the Department, that all IBS patients admitted to the Department should be informed about the study. A physical lecture was also held for a local working group within the Region Skåne, working with the implementation of healthy lifestyle habits.

*Announcements*

An information letter was sent to all primary healthcare centers (PCC) in the region (n=203). The letter contained study information and several study leaflets. The leaflets contained brief written information about the study, including contact details to the investigators. The staff at the centers were encouraged to distribute the leaflets in waiting rooms.

A contract was signed with Trialy AB, Gothenburg, Sweden to recruit participants through social media from Region Skåne. A questionnaire was constructed in which the interested individuals had to reply to a few questions to test suitability for entrance into the study ( Table 1). If individuals were not suitable according to any of the questions, they were automatically excluded from the system. However, they were able to complete the questionnaires as many times as they wanted. Two recruitment periods were performed: one during the summer/autumn of 2022, and one during the winter of 2022.

**Table 1.** Questionnaire on the digital announcement.

Question	Inclusion
Are you between 18-70 years?	**Yes** No
Have you experienced abdominal pain at least once weekly the last months	**Yes** No
Have you got the diagnosis IBS of any doctor?	**Yes** No
Do you eat any of the following diets: gluten-free, LCHF, low FODMAP or vegan food?	Yes **No**
Do you have more than two food allergies?	Yes **No**
Have you undergone major abdominal surgery	Yes **No**
Do you have any of the following diseases?	Heart-Lung diseases, diabetes, rheumatological disease, neurological disease, blood disease, malignancy, nephrological disease **Nothing of the above**
Are you pregnant or breast-feeding?	Yes **No**
Can you speak and write the Swedish language?	**Yes** No
Are you willing to adhere to a diet for the 4 weeks you participate in the study?	**Yes** No
Do you live in the Region of Skåne?	**Yes** No
I understand that by completing the form I am declaring my interest in participating in the study	**I see**

The correct answers are in bold. If the participant did not fulfill any of the criteria, the questionnaire was closed.

*Personal invitations*

A data search was performed in March 2022 at the Clinical Studies Sweden-Regional node in Southern Sweden. The search was performed on all medical records from the County of Skåne using the International Classification of Diseases (ICD) revision 10 to identify patients who had received any of the diagnoses K58.1, K58.2, K58.3, and K58.8 during 2019–2022. Initially, an information letter was sent randomly to the patients, and after a couple of weeks, the subjects were asked whether they were interested in participating. Because of the time-consuming routine with a few replying to the phone call and a few who wanted to participate, the information letter was changed, and the subjects were encouraged to call or email the investigators whether they were interested in participating in the study.

*Estimation of sample size*

The power calculation was based on non-inferiority, where a new treatment for IBS (SSRD) was tested against standard treatment (low FODMAP). The primary outcome was the responder rate (RR = ∆Total IBS-SSS ≥ −50), which was assumed to be 65% in both treatment groups. A difference in responder rate as large as 20% in favor of the standard treatment low FODMAP would allow the new treatment SSRD to be non-inferior. The sample size was based on 80% power, a one-sided confidence level of 97.5%, and an expected loss to follow-up of 10% to confirm non-inferiority was calculated to be 100 patients in each group. Since very few of the included patients were lost to follow-up, a new contact was made with the statistician. After discussion, the study was concluded in September 2023, although only 155 and not 200 patients were included.

## Results

### Recruitment processes

*Referrals from healthcare staff*

**Figure 1.**
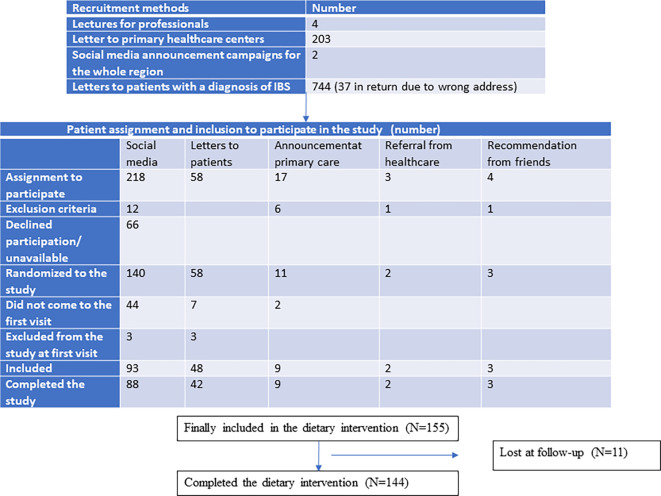
Flow chart over the recruitment process.

One written referral from a private healthcare center and two phone call referrals from the Department of Gastroenterology were obtained. One patient from the Department of Gastroenterology had bile acid malabsorption, but the other two were eligible for inclusion ( Figure 1).

*Self-referrals from patients*

Eight patients contacted the investigators because they were encouraged to participate by a dietician or GP at the PCC. Three of these were excluded because they fulfilled any of the exclusion criteria for the study ( Figure 1). Nine patients contacted the investigators because of information leaflets in the waiting room at their PCC, and four patients after recommendations from friends or relatives. Some patients fulfilled any of the exclusion criteria ( Figure 1).

*Advertisements in social media*

Two different recruitment campaigns on social media were performed by the professional company Trialy. The contract was to recruit 100 patients at each campaign, who were willing to participate in the study and who fulfilled the inclusion criteria without any of the exclusion criteria. The 218 assigned patients were contacted by phone by one of the investigators (BR). Upon calling, 66 participants did not answer or declined to participate, whereas 12 fulfilled any of the exclusion criteria. After exclusion, 140 patients (64.2%) were willing to participate in the study ( Figure 1).

*Medical records*

From the medical records, 8999 IBS patients were identified from the whole county, and 3587 patients in the age group of 18–70 years in the area close to Malmö. Of these 3587 cases, 744 were randomly selected and contacted through a written letter to be informed about the study and contact details to both investigators. Of them, 58 (7.8%) were willing to participate in the study.

### Randomization and inclusion processes

Patients who were willing to participate in the study were randomized to either the low FODMAP or SSRD group by block randomization. After randomization, 53 patients did not attend the first meeting. After new contact, they declined participation if they answered the phone call. Three patients were excluded at the first visit because they did not fulfill the inclusion criteria of a total IBS-SSS score of 175, one suffered from cognitive failure, one suffered from severe heart disease with bile salt malabsorption, and one suffered from microscopic colitis. Finally, 155 patients with IBS (72.4% of randomized cases) underwent dietary intervention ( Figure 1). This means an inclusion rate of 42.7% in the group recruited from social media and 6.5% in the group recruited from the medical records. ^18^ Reporting guidelines for the study are available. ^19^

### Lost at follow-up

Eight participants interrupted the intervention because they thought it was too difficult to adhere to the diet or because the symptoms were exaggerated by the diet. Of these, four were recruited from social media and four from medical records. One participant interrupted the intervention due to acute diverticulitis, one due to personal troubles, and one did not start the dietary intervention ( Figure 1).

### General comments

During the consultations with the patients, both during the recruitment and inclusion processes, it was found that many patients had low knowledge about nutrition, the benefits of a healthy diet, or the risks associated with malnutrition. These dialogues were not formally structured or analyzed.

## Discussion

The main finding in the present study was the great challenge in motivating patients with IBS to participate in a dietary intervention, although all patients were offered one of two efficient diets. ^12^^,^^13^

Several systematic reviews and meta-analyses have shown that a low FODMAP diet is efficient in IBS, with a marked improvement in global and specific gastrointestinal symptoms. ^20^^,^^21^^,^^22^ However, quality of life has not improved in parallel with symptom reduction. ^22^^,^^23^ The reason for unaffected quality of life may be the symptom burden of comorbidity and necessity of food avoidance. ^6^^,^^20^^,^^23^ Despite evidence of the beneficial roles of dietary modifications in several studies, it is a great adjustment for individuals in daily life to change dietary habits. This may have an impact not only on themselves but also on their families, friends, working routines, and social life. It costs a lot of motivation to make pervasive changes to daily routines. Adherence to a strict diet may have equal or more adverse effects on their lives than bowel symptoms. ^23^

Poor dietary intake of IBS has been reported in several studies, with a lower nutritional intake than recommended. ^15^^,^^24^ This may have been due to dietary changes and food avoidance. However, SSRD reduced the symptoms in parallel with healthier dietary habits, ^13^^,^^15^ which was not observed after low FODMAP. ^25^ Thus, food avoidance does not necessarily mean impaired nutrient intake. Since dietary risk factors largely contribute to diseases and death worldwide, ^25^ work to support and improve the dietary habits of the population is extremely important to reduce the disease burden. ^26^

Several studies have demonstrated that a high proportion of the population does not engage in health-promoting behaviors. ^27^ This may be partly due to the lack of knowledge about the consequences of different lifestyle habits. The great disease burden of IBS on the healthcare system, ^7^^,^^8^ in parallel with the difficulties in motivating patients to change their lifestyle habits to a healthier diet, rendering fewer symptoms, ^20^^,^^21^^,^^22^^,^^27^ is a challenge for the system. Although healthcare centers were informed about the study through lectures and written letters, only a few referrals came from this resource. This may be because physicians and dieticians informed about the study, but the patients declined the opportunity for referral. Several methods have been used to increase awareness of healthy behaviors. Motivating interviewing is a method practiced in primary healthcare, which may be useful for achieving behavioral changes. ^26^^,^^28^ It is also important to recommend dietary regimes that are simple to follow with a minimum of interference with daily life to increase quality of life in parallel with reduced symptoms and health risks. Low FODMAP has been found more difficult to adhere to than other diets, which may be discouraging for patients. ^29^ A recommended diet should also consider economic factors. In qualitative studies, the participants stressed the importance of continuous support from both family, friends, and the healthcare system as well as individual dietary treatment strategies, which is not often provided in clinical trials or in the health care. ^30^^,^^31^ Furthermore, simple lists with general recommendations may be difficult to adhere to if the basic knowledge about food is limited, and different dietary recommendations may be found contradicting. ^31^ Patients should be more involved in the production of material and development of dietary activities. ^31^ More prospective studies of behavior in patients living with IBS is necessary to learn how to motivate and support patients to change their dietary behavior. ^32^

We conclude from the current study that announcements on social media were the most efficient recruitment process. ^18^ Still, more than one-third of the initially assigned patients were unwilling to participate when they were contacted by the investigator, and one-third of the randomized participants did not show up at the appointment for inclusion. Hiring a company for announcements on social media is expensive. However, it seems to be the most superior method for recruiting patients and is the least time-consuming for investigators. Furthermore, those included in this recruitment arm were motivated and interested in dietary habits. However, there is a risk that recruitment from social media attracts those who are already aware of dietary factors, and those who have the most benefits of dietary improvements are never included. When inviting patients randomly or consecutively, participants may be more representative of the patient group.

In conclusion, despite clear evidence of successful dietary programs, it is difficult to recruit IBS patients for dietary interventions/treatments. Announcement through social media seems to be the most efficient way to recruit patients for dietary interventions.

### Ethical considerations

This study was conducted in accordance with the Declaration of Helsinki. Thus, all reported research has been conducted in an ethical and responsible manner and is in full compliance with all relevant codes of experimentation and legislation. This study was approved by the National Swedish Ethical Review Authority ( https://etikprovningsmyndigheten.se/en/), 2021-05407-01, date of approval 10/11/2021. All participants provided written informed consent before participating in the study. The participants gave written consent for the inclusion of material pertaining to themselves in publications and acknowledged that they could not be identified via the paper.

## Authors contribution

BO and BR were responsible for study design and data acquisition. BO was responsible for statistical analysis, interpretation of data, and drafting of the manuscript. BO obtained funding. Both the authors contributed to the manuscript and approved the submitted version.

## Data Availability

Figshare: Underlying data for ‘Challenges of recruitment processes to a randomized dietary trial in irritable bowel syndrome’, https://doi.org/10.6084/m9.figshare.25562292. ^18^ Figshare: CONSORT checklist for ‘Challenges of recruitment processes to a randomized dietary trial in irritable bowel syndrome’, https://doi.org/10.6084/m9.figshare.25399030. ^19^ Data are available under the terms of the Creative Commons Zero “No rights reserved” data waiver (CC0 1.0 Public domain dedication).
