# Supplementary material for: Challenges of recruitment processes to a randomized dietary trial in irritable bowel syndrome
Source: F1000Res. 2024 Jun 24;13:323. Originally published 2024 Apr 23. [Version 2] doi: 10.12688/f1000research.147710.2 (PMC11208853; doi:10.12688/f1000research.147710.2)
Supplement: Peer review history

# Reviewer report: Reviewer response for version 2

**DOI:** 10.5256/f1000research.168054.r294855

**Published:** 2024-06-26

**Authors:** Jari Punkkinen

**Affiliations:** 1 Helsinki University Central Hospital, Helsinki, Finland

The authors have successfully answered all my concerns and I find the article suitable for indexing.

Is the work clearly and accurately presented and does it cite the current literature?

Yes

If applicable, is the statistical analysis and its interpretation appropriate?

Yes

Are all the source data underlying the results available to ensure full reproducibility?

Yes

Is the study design appropriate and is the work technically sound?

Yes

Are the conclusions drawn adequately supported by the results?

Yes

Are sufficient details of methods and analysis provided to allow replication by others?

Yes

Reviewer Expertise:

Functional gastrointestinal disorders

I confirm that I have read this submission and believe that I have an appropriate level of expertise to confirm that it is of an acceptable scientific standard.

# Reviewer report: Reviewer response for version 1

**DOI:** 10.5256/f1000research.161936.r283692

**Published:** 2024-06-14

**Authors:** Tarek Mazzawi

**Affiliations:** 1 Al-Balqa Applied University, Al-Salt, JOR, Jordan

This research describes the challenges the researchers face while recruiting patient with IBS into research studies. This is a very important issue both for medical researchers and reviewers who demand high power and large study cohorts to understand that IBS patients are not easy to recruit. 

 I have minor comments:

 1) Under Methods- personal invitations: please add the coding system used for setting the diagnosis : ICD 10 

 2) Under discussion: 

 The authors seem to drift from the main topic (challenges of recruitment processes) while writing in the discussion (the 3rd paragraph) about the different diets and low vitamin D supplements and their relations to IBS symptoms. The point is to discuss why IBS patients drop out, factors that influence their acceptance or withdrawal from the studies, any psychological issues that affect their motivations ,,etc.  Also if there are solutions that can be suggested to solve these challenges . I suggest that the discussion should be edited to cover these issues.

Is the work clearly and accurately presented and does it cite the current literature?

Yes

If applicable, is the statistical analysis and its interpretation appropriate?

Yes

Are all the source data underlying the results available to ensure full reproducibility?

Yes

Is the study design appropriate and is the work technically sound?

Yes

Are the conclusions drawn adequately supported by the results?

Yes

Are sufficient details of methods and analysis provided to allow replication by others?

Yes

Reviewer Expertise:

Assistant Professor, MD, PhD, Gastroenterologist and Hepatologist. My research involves: IBS, microbiota, fecal microbiota transplantation, intestinal ultrasound, endoscopy and sedation.

I confirm that I have read this submission and believe that I have an appropriate level of expertise to confirm that it is of an acceptable scientific standard.

# Response

**Published:** 2024-06-17

**Authors:** Bodil Ohlsson

**Affiliations:** Clinical Sciences, Lund University, Malmö, Sweden

**Tarek Mazzawi**, Al-Balqa Applied University, Al-Salt, JOR, Jordan 

**Comment:** This research describes the challenges the researchers face while recruiting patient with IBS into research studies. This is a very important issue both for medical researchers and reviewers who demand high power and large study cohorts to understand that IBS patients are not easy to recruit. 

**Reply**: Dear Reviewer, thank you for evaluating this manuscript. We do appreciate the constructive criticism given and have tried to answer the questions. All changes are marked as tracked changes. We think that the changes have improved the manuscript.

 On behalf of both authors,

 Bodil Ohlsson, Professor, Senior Consultant of Gastroenterology and Hepatology

**Comment:**I have minor comments:

 1) Under Methods- personal invitations: please add the coding system used for setting the diagnosis : ICD 10 

**Reply**: This has now been added on page 5, last section.

**Comment: **2) Under discussion: 

 The authors seem to drift from the main topic (challenges of recruitment processes) while writing in the discussion (the 3rd paragraph) about the different diets and low vitamin D supplements and their relations to IBS symptoms. The point is to discuss why IBS patients drop out, factors that influence their acceptance or withdrawal from the studies, any psychological issues that affect their motivations ,,etc.  Also if there are solutions that can be suggested to solve these challenges . I suggest that the discussion should be edited to cover these issues.

**Reply:** You are completely right, and now we have changed the discussion accordingly. Hope you find this better and more relevant. See page 8, third section.

# Reviewer report: Reviewer response for version 1

**DOI:** 10.5256/f1000research.161936.r272869

**Published:** 2024-05-29

**Authors:** Jari Punkkinen

**Affiliations:** 1 Helsinki University Central Hospital, Helsinki, Finland

This is an interesting study about the difficulty of recruiting IBS patients to a dietary trial. I have only some concerns.

 The abstract background describes that this paper about the recruitment process is part of a larger randomized study, about FODMAP and SSRD in IBS patients. It would be clarifying to tell in the abstract that the results of the dietary trial itself are not included in this paper but will be presented in another publication. 

 In the methods section the authors tell that questionnaires, diary books, blood samples and fecal samples were collected as part of the dietary trial. It would be useful to describe these more in detail because if this caused a lot of effort and work for the patients, it might have hindered patients from participating in the study. 

 It would also be important to tell, how the FODMAP and SSRD diets were carried out. Were the patients provided the food they ate or did the patients only receive instructions how to prepare their meals and what food to buy ? Providing ready made meals is easier than only giving instructions and orders to prepare meals. Buying food to prepare a FODMAP/SSRD diet might also result expensive. All this might hinder patients from participating in the study. 

 What did the FODMAP/SSRD diets consist of ? People have different tastes and not all are willing to commit to a restricting diet for four weeks. This should be described briefly

 is is an interesting and important finding regarding future studies that the easiest way to recruit patients for a dietary trial is through the social media.

Is the work clearly and accurately presented and does it cite the current literature?

Yes

If applicable, is the statistical analysis and its interpretation appropriate?

Yes

Are all the source data underlying the results available to ensure full reproducibility?

Yes

Is the study design appropriate and is the work technically sound?

Yes

Are the conclusions drawn adequately supported by the results?

Yes

Are sufficient details of methods and analysis provided to allow replication by others?

Yes

Reviewer Expertise:

Functional gastrointestinal disorders

I confirm that I have read this submission and believe that I have an appropriate level of expertise to confirm that it is of an acceptable scientific standard, however I have significant reservations, as outlined above.

# Response

**Published:** 2024-06-17

**Authors:** Bodil Ohlsson

**Affiliations:** Clinical Sciences, Lund University, Malmö, Sweden

**Jari** **Punkkinen**, Helsinki University Central Hospital, Helsinki, Finland 

**Comment**: This is an interesting study about the difficulty of recruiting IBS patients to a dietary trial. I have only some concerns.

**Reply**: Dear Reviewer, thank you for evaluating this manuscript. We do appreciate the constructive criticism given and have tried to answer the questions. All changes are marked as tracked changes. We think that the changes have improved the manuscript.

 On behalf of both authors,

 Bodil Ohlsson, Professor, Senior Consultant of Gastroenterology and Hepatology

**Comment**: The abstract background describes that this paper about the recruitment process is part of a larger randomized study, about FODMAP and SSRD in IBS patients. It would be clarifying to tell in the abstract that the results of the dietary trial itself are not included in this paper but will be presented in another publication. 

**Reply**: Thanks for this comment that will make the intention of the publication much clearer. This is now added in both the abstract and in the last sentence of background, page 3.

**Comment**: In the methods section the authors tell that questionnaires, diary books, blood samples and fecal samples were collected as part of the dietary trial. It would be useful to describe these more in detail because if this caused a lot of effort and work for the patients, it might have hindered patients from participating in the study. 

**Reply: **The number of physical visits as well as the time needed for each visit and completion of questionnaires are now given along with more details about the study design, page 3, second section. A new reference is added for IBS-SSS, ref No 16, which was unfortunately forgotten in the first version.

**Comment**: It would also be important to tell, how the FODMAP and SSRD diets were carried out. Were the patients provided the food they ate or did the patients only receive instructions how to prepare their meals and what food to buy ? Providing ready made meals is easier than only giving instructions and orders to prepare meals. Buying food to prepare a FODMAP/SSRD diet might also result expensive. All this might hinder patients from participating in the study. 

**Reply**: The participants received oral and written information about the food. They had to buy and cook all the food themselves, but they received menu suggestions and recipes to make it easier for them, page 4, first section. However, since many people buy ready-to-eat food it was a great challenge for many of them to plan and cook the food on their own. It could also be more expensive to buy more fruits, vegetables and fish. On the other hand, to cook the food themselves could be cheaper than to buy food ready-to-eat, soda, candies and go to restaurants. This is now discussed in the discussion, page 8, third section.

**Comment**: What did the FODMAP/SSRD diets consist of ? People have different tastes and not all are willing to commit to a restricting diet for four weeks. This should be described briefly

**Reply:** The diets are now briefly described on page 4, first section. However, since this was only a methodological study about the recruitment process, we have kept this information short.

**Comment**: It is an interesting and important finding regarding future studies that the easiest way to recruit patients for a dietary trial is through the social media.

**Reply**: Yes, we think so
